# iPSCs and Exosomes: Partners in Crime Fighting Cardiovascular Diseases

**DOI:** 10.3390/jpm11060529

**Published:** 2021-06-09

**Authors:** Giulia Germena, Rabea Hinkel

**Affiliations:** 1 Laboratory Animal Science Unit, Leibniz-Institut für Primatenforschung, Deutsches Primatenzentrum GmbH, Kellnerweg 4, 37077 Göttingen, Germany; 2 DZHK (German Center for Cardiovascular Research), Partner Site Göttingen, 37077 Göttingen, Germany; 3 Stiftung Tierärztliche Hochschule Hannover, University of Veterinary Medicine, 30559 Hannover, Germany

**Keywords:** heart failure, cardiovascular diseases, iPSCs cardiomyocyte, platforms, cardiopatches, exosomes, innate immunity, 2D and 3D models

## Abstract

Cardiovascular diseases are the leading cause of mortality worldwide. Understanding the mechanisms at the basis of these diseases is necessary in order to generate therapeutic approaches. Recently, cardiac tissue engineering and induced pluripotent stem cell (iPSC) reprogramming has led to a skyrocketing number of publications describing cardiovascular regeneration as a promising option for cardiovascular disease treatment. Generation of artificial tissue and organoids derived from induced pluripotent stem cells is in the pipeline for regenerative medicine. The present review summarizes the multiple approaches of heart regeneration with a special focus on iPSC application. In particular, we describe the strength of iPSCs as a tool to study the molecular mechanisms driving cardiovascular pathologies, as well as their potential in drug discovery. Moreover, we will describe some insights into novel discoveries of how stem-cell-secreted biomolecules, such as exosomes, could affect cardiac regeneration, and how the fine tuning of the immune system could be a revolutionary tool in the modulation of heart regeneration.

## 1. Introduction

Cardiovascular diseases (CVDs) are the number one cause of death globally; more people die annually from CVDs than from any other cause (source WHO).

On the basis of the 2017 National Health Interview Survey, about 17.8 million deaths were attributed to CVDs worldwide, which amounted to an increase of 21.1% from 2007 [1].

Heart failure is a direct consequence of the intrinsic inability of damaged cardiomyocytes to be replaced. While cardiomyocyte turnover is high in early childhood, in adulthood it decreases to only 1% [2]. When the injured myocardium has to be replaced, fibroblasts take the place of myocytes, leading to the formation of fibrotic tissue. This so-called tissue remodeling induces typical pathological features, such as cardiac hypertrophy and dilatation of the chambers, which finally lead to the progression of heart failure [3].

Traditional CVD treatments focus on slowing the progression of the disease or on ameliorating already existing damage. To date, no methods are available to reverse CVDs. Cardiac assist devices, which provide mechanical circulatory support, stabilize the cardiac functionality of the patient while on the waiting list for heart transplantation [4]. This so called “bridge to transplant” approach lasts two years on average; therefore, heart transplantation is currently the only existing possibility of long-term treatment for end-stage patients. Unfortunately, this approach has a high impact on health costs, and is limited by the low number of donors [5].

The need for alternative therapies able to reverse cardiovascular damage is therefore compelling. One of the major difficulties in the design of new therapies arises from the high complexity and the presence of multiple entities leading to CVDs. While small animal models are a powerful tool for basic research, allowing a deep and detailed observation of specific traits of a certain disease, the translation to the human system often fails to resemble the phenotype observed in the animal model. A huge number of compounds/targets that are highly effective in cell culture systems or in rodents, for example, fail once tested in clinical trials. In particular, non-human models cannot recapitulate the complete human pathophysiology of such a variable disease. For example, when we compare a mouse and a human heart, resting heart rate is 10 times higher in mice; in addition, responses to exercise and arrhythmias are different. Moreover, the discrepancy in terms of ion channel expression and pharmacokinetics mandates careful interpretation of results before they are extrapolated to humans [6]. An example of these differences has recently been published. Vegter et al. were not able to detect the typical miRNA downregulation observed in patients when analyzing well-established rodent heart failure models, highlighting once more the discrepancy between human and rodent models [7].

An additional important element to take into account while studying CVDs is the influence of comorbidities and risk factors such as the presence of diabetes, hypertension, and unhealthy lifestyle. These features play a key role in the development of CVDs, and while it is not possible to fully recapitulate these elements in rodent models, large animal models (pigs, sheep, dogs, and non-human primates) provide some of these characteristics, representing a bridge between rodent and humans. The efficiency of clinical translation from large animal models to humans has already been extensively demonstrated [8]. It is important to note that, while large animal models are suitable for therapeutic studies, the analyses of detailed and fine molecular mechanisms are laborious. It is possible to conclude that, while rodents are the primary choice for the dissection of the specific molecular mechanisms driving pathology onset and progression, large animal models are needed for the investigation of possible therapeutic approaches.

Human-based in vitro models represent the perfect compromise. On one hand they present the potentiality for the study of therapeutic approaches, and on the other hand the possibility of exploring complex molecular pathways.

In 1998, the potential of pluripotent human embryonic stem cells (ESCs) has been described [9]. However, due to legal and ethical issues, there are multiple limitations on the use of ESCs. In the search to overcome these limitations, other approaches developed. Since their discovery in 2006, induced pluripotent stem cells (iPSCs) immediately stand out for their potential [10]. While their differentiation capabilities are similar to those of ESCs, their somatic origin does not raise the same ethical questions as ESCs. In the cardiovascular field, the first studies of iPSCs’ reprogramming of cardiomyocytes were conducted on murine cells [11], and we had to wait until 2009 to obtain functional cardiomyocytes derived from human iPSCs [12].

The era of human iPSC cardiomyocytes broke new ground in cardiovascular research. While on one hand there is the potential of substituting animal experiments, and of generating models or platforms mimicking different CVD pathologies in order to evaluate the effects of distinct compounds; on the other hand, the possibility of “exchanging” or repairing the damaged cardiomyocytes with new ones is astonishing.

In this review we want to give an overview of recent developments in disease modelling and treatments using iPSCs, with special emphasis on the promising potential of iPSC-derived exosomes and the role of immune system modulation.

## 2. Platforms for the Analysis of Cardiovascular Diseases

The availability of models to study molecular mechanisms and diseases is a basic prerequisite to developing a cure. In the cardiovascular field, the absence of well-established disease models led to a generally poorly developed pool of therapies. A precise, patient-specific treatment could be developed only on the basis of a model that finely recapitulates, on a molecular level, the onset and progression of the pathology, preferably as close as possible to the human situation.

The discovery of iPSCs’ reprogramming of cardiomyocytes provided the first tool in the approach to the deep analysis of CVDs [13]. In recent years, 2D models were largely used not only as an inexpensive method for high-throughput screening of chemical libraries of compounds but, more importantly, as specific tools to study different CVD mechanisms. In particular, iPSCs derived from patients suffering from genetic-driven CVDs (Mendelian CVDs) were able to display specific pathology-related alteration. For example, the typical cellular hypertrophy with abnormal sarcomere organization and altered calcium signaling were described in cells generated from patients with hypertrophic cardiomyopathy bearing a mutation in the *MYH7* gene [14]. A parallel study [15] obtained similar results generating cells bearing a different *MYH7* mutation. These two studies are just examples of the plasticity and the many possibilities offered by the iPSC 2D culture method. Other Mendelian CVDs, such as dilated cardiomyopathy (DCM), left ventricular non-compaction cardiomyopathy (LVNC), and arrhythmogenic cardiomyopathy (ACM), have been shown to be phenotypically reproduced in vitro [16,17,18,19]).

After the proof that cardiac iPSCs obtained from a patient bearing a specific genotype were able to recapitulate the CDV phenotype in vitro, the subsequent step was the genetic manipulation of iPSCs. Taking advantage of the CRISPR/Cas9 technology, it is possible to replace the mutated gene with its wild-type form in order to rescue the defect induced by the mutation [20]. In addition, the possibility of having access to iPSCs bearing a specific mutation could be problematic; therefore, the introduction of a particular mutation via genome editing is a powerful tool for the study of rare genetic pathologies [21]. This approach also brings some advantages compared to the generation of iPSCs from patients, especially when it comes to the analysis of the pathological molecular mechanism. One major concern during this type of study is the number of cellular lines used for the analysis. The screening of multiple lines is needed to verify whether a mutation leads to a particular phenotype; the consensus is that at least three different iPSC lines derived from three different patients need to be compared against three lines derived from three healthy donors. The genetic background, the difference in the source, and the cultivation methods render the line comparison demanding. The generation of isogenic pairs, via the insertion/deletion of mutations in healthy iPSCs by genetic manipulation, allows scientists to determine the effect of defined manipulation by eliminating all other external parameters. For additional information regarding this topic, a comprehensive analysis has been performed by Musunuru et al. [22].

In addition to the potentialities in the analysis of the molecular mechanism driving different pathologies, iPSCs also offer a powerful tool for the therapeutic evaluation of different compounds in a specific cohort of patients. For example, the screening of a family affected by long QT syndrome not only allowed the identification of a precise mutation driving the pathology, but also showed that iPSCs derived from different family members were able to perfectly recapitulate the electrophysiological features of the disorder [23]. These findings indicate these cells to be a tool for the characterization of the disease, and for the study of possible therapeutic approaches of cohort of patients bearing this specific mutation. With this method, it would be possible to specifically evaluate the efficacy of multiple compounds and different dosing on cells presenting distinctive characteristics of the patient, thus guaranteeing the safety of the treatment. A deeper genomic analysis of multiple iPSC lines derived from different subjects affected by the same pathology would lead to a comprehensive determination of the distinctive mechanism that induces a particular phenotype. The stratification of the subjects on the basis of the same genomic mutation could contribute to the generation of specific treatment established by the subtype classification.

The use of iPSCs for drug discovery raises the question of cardiomyocyte maturity. In vivo, the maturation of cardiomyocytes requires the presence of specific mechanical and electrical factors in addition to biochemical stimuli and cellular interaction. The absence of these factors in vitro leads to relatively immature cardiomyocytes that, together with a small cellular size, display a reduced electrical potential and an incomplete excitation–contraction coupling [24].

To optimize iPSCs’ differentiation of mature cardiomyocytes, efforts have been made to mimic the physiological environment surrounding a developing cardiomyocyte in vivo. For example, in a 2D system, the co-culture of cardiomyocytes with other cell types, such as endothelial cells, has been shown to enhance their maturation [25]. Co-culture is only one of the multiple possible approaches. Comparison in terms of cardiomyocyte maturation on substrates with different stiffness demonstrates the importance of the mechanotransduction stimulus during cardiomyocyte differentiation [26]. In addition to matrix rigidity, mechanical stimulation—such as substrate stretching—plays a role in cell maturation [27]. Despite the optimization of these protocols, the absence of a physiological environment is still a big limitation. In order to mimic physiological microenvironment signaling, in recent decades, new in vitro approaches with organoids and engineered heart tissue have been developed. In particular, an ideal 3D model is composed of three components: (1) cardiomyocytes surrounded by (2) endothelial and stromal cells on (3) an extracellular matrix ECM. While endothelial and stromal cells support cardiomyocyte differentiation and structure [28,29], the ECM network composed of perimysial collagen fibers connects the cardiomyocytes, provides a basic structure, creates stress–strain, and allows for the anisotropic alignment of the cardiomyocytes [30]. The advantage of using a 3D system is the possibility of assessing functional parameters, such as contraction or electrical properties, as in a whole tissue, mimicking the physiological situation as close as possible. One of the first studies applying this technology focused on the analysis of DCM. Hinson et al. demonstrated that iPSC cardiomyocytes bearing different titin mutants display a defect in contractile function, recapitulating the patient phenotype [19]. Interestingly, the same analysis in a 2D iPSC model was not able to show the typical DCM phenotype.

The potential of a 3D system provides not only the possibility of studying the pathological mechanisms related to genetic diseases, but also the possibility of analyzing non-genetic-dependent medical conditions, such as tissue remodeling or arrhythmia. Different research groups were able to mimic typical features of remodeling following myocardial infarction—such as loss of cardiomyocyte viability, calcium handling, and fibrosis—in vitro [31,32]. These models will enable the screening of drugs for the treatment of heart damage.

Myocardial infarction is not the only model developed from a 3D culture method. Tachypacing of 3D culture is a powerful tool for the study of antiarrhythmic drugs [33]. The potentiality of these models is outstanding, but the complex and expensive equipment, as well as the high degree of knowhow needed to perform 3D cultures, are limits to broad application. However, in the era of further implementation of the 3R rules (replacement, reduction, and refinement of animals in experiments) and the continuous research of new methods to try to reduce or replace animal experiments, incorporating the use of these methods as a first step for cardiotoxicity screenings, where a large number of compounds have to be tested, represents a promising potential strategy to reduce animal numbers. The analysis of the synergic effects in a multiorgan setting is nevertheless crucial in drug development; it is thus unlikely that animal models will be completely eliminated, at least in the short term. In 2020, the first integrated multiorganoid body-on-a-chip system was tested. The authors were able to prove the toxicity of drugs recalled by the FDA due to their adverse effects on humans with their 3D multiorganoid approach [34]. A better predictive model will increase the success of clinical trials, reduce costs, and enhance drug safety.

## 3. 2D, 3D, and Engineered Cardiac Tissue as Therapeutic Treatment

2D and 3D in vitro models are powerful tools as screening platforms for novel potential drugs/therapies, and for the study of mechanical/genetic models driving pathological conditions. The effects of a specific drug could be efficiently evaluated in vitro. These features are already quite outstanding, and in recent years the idea of using these models as therapeutic approach to block and repair cardiac remodeling has become popular.

The first cellular approach was performed using mesenchymal stem cells (MSCs), for their capability to differentiate and to secrete paracrine effectors [35,36]. MSCs were shown to stimulate proliferation and differentiation [37], and to display an antifibrotic effect. More recent approaches were performed using embryonic stem cells (ESCs). The groups of Laflamme and Murry focused their efforts on the characterization, in multiple animal models, of hESC cardiomyocyte grafts, and their effects on heart remuscularization and arrhythmia onset. The analysis of single-cell hESC cardiomyocyte transplantation in the infarcted region of immunocompromised rats showed improved cardiac function, at least in a short-term (4 weeks) follow up [38]. However, despite a short-term engraftment, these positive effects were lost when performing long-term (12 weeks) follow up [39]. The performance of the same experiment in large animal models provided some controversial results. Even though the positive effects on tissue regeneration and the improvement in mechanical function were observable in injured guinea pigs hearts [40] and infarcted pig hearts, in this last model, episodes of ventricular tachyarrhythmias were detected, raising the question of the safety of this type of treatment [41]. In a non-human primate model of myocardial ischemia–reperfusion, hESC cardiomyocytes present grafts perfused by host vessels, but, as detected in infarcted pigs, arrhythmia events were recorded [42]. In accordance with these findings, another study demonstrated that hESCs’ intracardiac injection in non-human primates with myocardial infarction leads to cardiac improvement and graft integration, even though a subset of animals experienced graft-associated arrhythmias [43]. The precise mechanism of stem-cell-mediated heart regeneration is still debated [44,45]. Some studies, performed in mice and non-human primates, claim that the tissue repair mediated by single-cell hiPSCs is mainly due to paracrine effects and not to cellular engraftment, since engraftment is observable only for a short time period after transplantation [46,47]. To overcome short-term viability, and to ameliorate cellular engraftment, 3D systems have been generated. The analysis of the implantation of cardiac cell sheets [48] or administration of hESC cardiomyocyte aggregates [49] demonstrated an enhanced survival and high engraftment when compared to single-cell injections. It is possible to distinguish two major types of 3D system on the basis of the absence or presence of external scaffolds. In particular, while organoids are structures generated by cellular self-organization, engineered tissues are characterized by the presence of non-cellular materials. Due to their multicellular composition, which recapitulates the interconnection of different cell types and their tissue-like structures, organoids are perfect models for molecular studies, but still fail to resemble the high complexity of the heart. For this reason, tissue engineering is becoming the principal choice for therapeutic approaches. The presence of an external scaffold provides physical/mechanical support, enables cardiomyocyte maturation and, conversely to single-cell injection—which shows diffusion and poor cellular retention—the engineered tissue allows for stable and precise application. For these reasons, in recent years, the generation of cardiopatches is becoming an attractive technique in the therapeutic area. From the development of the first cardiopatch, obtained by mixing cardiomyocytes and collagen [50], huge steps have been taken in the characterization of scaffold materials and in the analysis of the combination of cardiac and non-cardiac cellular components in order to recapitulate the heart’s physiological composition. Approximately a decade after the first cardiopatch characterization, Menasché et al. [51] demonstrated, for the first time in a human patient, that hESC cardiac progenitor cells, embedded in a fibrin scaffold, were able to restore heart functionality. This study set a milestone for cardiopatch development. From this starting point, different materials, scaffold shapes, and cell compositions have been used to obtain the most performant cardiopatch, not only in terms of heart functionality, but also in terms of engraftment. A study of Narita et al. showed that cardiopatches’ xenotransplantation in infarcted rat hearts improved survival rates and led to an improvement in angiogenetic functions [52]. After this study, subsequent analysis in both mice and rats showed that cardiopatches were able to ameliorate endothelial functions and to significantly increase survival [53,54]. In the same timeframe, a follow-up study from Menasché et al. [55] was published. The authors analyzed the feasibility, safety, and efficacy of hESC-derived cardiovascular progenitor patches implanted in six patients during a coronary artery bypass procedure. No adverse events regarding teratoma formation, arrhythmia, or alloimmunization were observed during the one-year follow up. Even though all patients presented cardiac function improvements, unfortunately, due to the small sample size and the concomitant coronary artery bypass, the real efficacy of the treatment could not be fully estimated, and additional data need to be collected and evaluated. In parallel to Menasché’s studies, Zimmermann et al. focused their efforts on generating ESC- and iPSC-engineered human myocardium with a good manufacturing practice protocol—a key requirement for a clinical translation step [56]. During their study, the researchers were able to achieve a milestone in the generation of cardiopatches suitable for human implantation. In particular, they: (1) optimized and standardized cardiomyocyte maturation by specific co-culture composition (70%/30% cardiomyocyte/fibroblast), (2) developed a specific cardiomyocyte medium cocktail able to enhance cell viability, (3) established a serum-free protocol based on medical-grade bovine collagen instead of Matrigel, and (4) scaled up cardiopatch production to a clinically relevant level. The establishment of a GMP process for clinically applicable cardiopatch generation (Patent EP2840132B1), and the confirmation of the safety of implantation in terms of tumor formation and arrhythmogenesis, established the background for the application of a clinical trial. In December 2020, the clinical trial “Biological Ventricular Assist Tissue in Terminal Heart Failure”—BioVAT-HF-DZHK20—was started (NCT04396899). In this trial, 53 patients with terminal heart failure will receive a cardiopatch via thoracotomy, and their heart functionality will be evaluated.

## 4. Immunity Doubts

The generation of patient-specific iPSCs as source of regenerative cells and for pharmacological therapy evaluation displays broad potentialities. However, iPSC applications face a number of limitations, such as the time and costs necessary to generate patient-specific iPSCs. To overcome these limitations, the idea of generating off-the-shelf, universally transplantable iPSCs is growing, and the scientific community is working on the generation of iPSCs that minimize/eliminate immunological compatibility issues.

HLAs (human leucocyte antigens), encoded by the human MHC (major histocompatibility complex), are surface proteins that regulate the immune response. One of the major HLA functions is to present non-self peptides to lymphocytes (T cells). Once activated, T cells react against the non-self peptides and eliminate/reject them from the body. While this mechanism is pivotal in disease defense, it is also one of the major causes of organ transplant rejection. Immunosuppressant drugs need to be repeatedly administered in order to avoid organ rejection. At the same extent, it has been showed that in order to promote the engraftment and survival of allogenic MHC-matched cardiomyocytes in *Macaca fascicularis*, clinically relevant doses of immunosuppressant are needed [57]. These data indicate that in order to eliminate organ/cell rejection, the genome editing or elimination of the HLA complex is necessary. In this perspective, a recent study [58] showed that the complete elimination of B2M (beta microglobulin 2M—suppressing the expression of HLA-I) or CIITA (MHC II transactivator—suppressing the expression of HLA-II) leads to a complete deletion of the HLA complex. Cardiomyocytes derived from these cells were not only able to induce low T-cell activation, but also to escape natural killer recognition—the first defenders recognizing non-self peptides after transplantation [59]. A following study, eliminating HLA-I and II but simultaneously overexpressing CD47 (an additional surface marker) showed that these edited iPSCs were able to fully evade immunorecognition in MHC-mismatched allogenic recipients [60]. The presence of a massive number of surface markers with multiple functions renders the research of the perfect combination a highly complex topic. In the future, genome editing guidelines should be optimized for the generation of universal and hypoimmunogenic iPSCs for stem cell therapy [61].

## 5. Mechanism of Action: Exosomes and Immunity Regulation

A comprehensive knowledge of the mechanisms driving stem-cell-mediated heart regeneration is missing. As mentioned in the previous paragraphs, three different hypotheses for the beneficial effects of stem cells have been postulated: (1) mechanical support of the myocardium, (2) paracrine effects, and (3) immune modulation.

As described before, one approach of intervention in a chronic model of myocardial infarction is cardiopatch implantation. Preliminary experiments analyzing the efficacy of cardiopatches in rodent models of chronic myocardial infarction demonstrated that cardiomyocyte engraftment rates do not translate into a significant functional heart improvement. In particular, the implantation of cardiopatches previously irradiated—and thus, only providing a mechanical support—was able to ameliorate heart functionality. Of note, the observable effect was less pronounced compared to the cardiomyocyte-loaded cardiopatches, indicating that cellular components play an additive role. These findings were consistent with a lack of electrical coupling of the xenograft, indicating that a cell-independent mechanism, such as mechanical stabilization, could elicit a therapeutic effect [62]. When analyzing long-term cardiomyocyte retention, 12 weeks after transplantation, the authors showed a cardiomyocyte survival rate of only 10%. These data indicate that even a small engraftment rate is enough to drive therapeutic heart remodeling. These effects could be the result of the combination of mechanical support, paracrine effects, and heart remuscularization.

We have already pointed out that the need for the study and generation of functional, differentiated cardiomyocytes derived from iPSCs arises from the necessity of developing therapeutic approaches for the treatment of patients in a late stage of the pathology, trying to postpone or fully eliminate the necessity of heart transplantation. While in late stages of the disease invasive therapies such as cardiopatch implantation seem to be the gold standard, in the early stages of heart failure a less invasive approach is desirable. In this context, understanding the mechanism at the base of stem cell heart regeneration mediated by microenvironment modulation and immune system regulation has become an attractive topic.

Stem cell population represents a wide source of factors, such as chemokines, exosomes, and miRNAs. These factors could have a microenvironmental impact, leading to heart healing without the involvement of a direct cellular interaction—a so-called “paracrine effect”. Some reports were able to prove that 72 h after stem cell intramyocardial injection it was already possible to show a cardioprotective effect, clearly not attributable to myocardial regeneration from donor cells [63,64]. The deep analysis of stem-cell-conditioned media showed that exosomes were putatively responsible for the reduction in infarct size after ischemia and reperfusion. This finding opened the door to a new approach in tissue injury repair [65]. The phase 1 trial CADUCEUS (Intracoronary cardiosphere-derived cells for heart regeneration after myocardial infarction) was initially focused on cardiosphere-mediated regeneration of the injured heart muscle [66]. Nevertheless, a deeper analysis of this study pinpointed that the regenerative effect was mediated by cell-derived exosomes. [67]. The authors demonstrated that, when inhibiting exosome production, the beneficial effects of the therapy were suppressed. In parallel, the authors were able to show that one specific microRNA enriched in the pool of exosomes—miR146a—was able to recapitulate some of the regenerative effects once administered in the injured heart. These findings lead to the question of whether there is a real necessity of the injection of stem cells, or whether it is enough to treat the patient only with factors derived from stem-cell-conditioned media—and if so, which factors or combination would provide the optimal therapeutic effect. The direct comparison between iPSCs and extracellular vesicles in a murine model of myocardial infarction demonstrated that both of the treatments improve left ventricular function, hypertrophy, and myocardial survival, albeit the effect mediated by the vesicles was greater. In addition, while iPSCs resulted in teratoma formation, vesicle injection was safer, indicating them as a potential alternative for therapeutic application [68]. In accordance with these findings, a recent paper showed that both iPSCs and exosomes embedded in hydrogel and injected into the pericardial cavity were able to promote cardiac repair in a porcine model of myocardial injury [69]. Exosomes can be derived from different cell populations, and their different paracrine effects are dependent on the distinctive content of the exosome vesicles, as extensively revised in [70]. For example, while exosomes derived from human mesenchymal stem cells are enriched with miR21a, and regulate oxidative stress and calcium homeostasis [71], exosomes derived from cardiosphere-derived cells are enriched in miR-181 and regulate inflammation [72]. In addition, the analysis of iPSC-derived exosomes indicates that miR-21 and miR-210 are responsible for attenuating myocardial injury [73].

From a mechanistic point of view, exosomes are not only able to exert an antiapoptotic effect on cardiomyocytes and proangiogenic activity on endothelial cells [74], but also regulate the immunological properties needed for myocardial healing.

**Figure 1:**
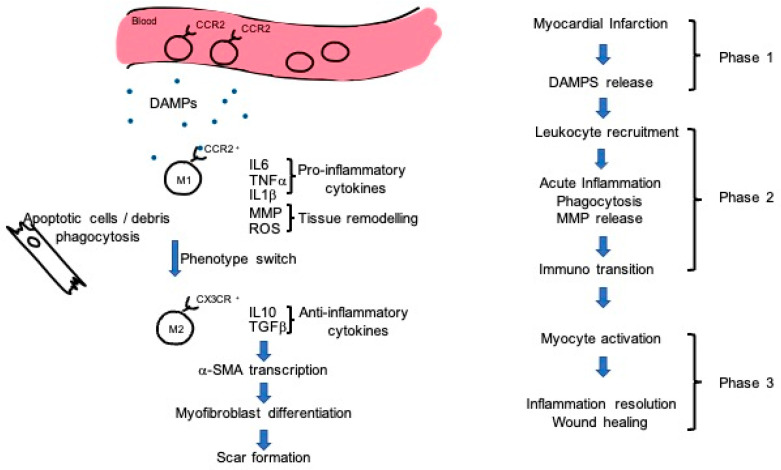
Different phases of immune activation during myocardial infarction. Cellular damage induces DAMPs and alarmin release, which lead to leukocyte infiltration. Once extravasated, macrophages—in particular the M1 subtype—release proinflammatory cytokines and metalloproteases (MMPs), which induce extracellular matrix remodeling. Cellular and debris phagocytosis influence macrophage phenotype switch. M2 subtype macrophages display an anti-inflammatory action, releasing IL-10 and TGF-β. This cytokine activates α-SMA transcription in fibroblasts that could differentiate in myofibroblasts and contribute to scar formation.

Analyzing the mechanisms driving stem cell effects during heart healing revealed a fundamental role of macrophages in this process [72,75]. In recent years, some clinical studies trying to ameliorate cardiac repair via reducing inflammation through immunosuppression failed to improve cardiac wound healing [76]. Rather than completely suppress the immune system, a fine regulation of the different immune cell populations could provide a therapeutic potential. During myocardial infarction, three different phases of immune system activation have been described. While in the first phase there is a release of alarmin and damage-associated molecular patterns (DAMPs), in the second phase (12–24 h post-infarction) there is a massive extravasation of leukocytes into the infarcted tissue, which leads to phagocytosis and the removal of apoptotic and necrotic cardiomyocytes [77,78]. In the third phase, the reparative phase, a subtype of macrophages and T lymphocytes promotes myofibroblast proliferation, neovascularization, and scar formation [79] (Figure 1).

It is important to point out that in response to myocardial infarction there are two different detectable subtypes of macrophages: M1 macrophages, with typical pro-inflammatory characteristics; and M2 macrophages, with anti-inflammatory properties. In their M1 status, macrophages clear the necrotic area, phagocytizing apoptotic cells. During this process, M1 macrophages polarize into M2 macrophages [80,81] that, accumulating in the infarcted area, exhibit reparative abilities after myocardial infarction. Depletion of the M2 population leads to impaired fibroblast activation and, in turn, a dramatic decrease in tissue repair [79]. Fine regulation of the macrophage population could be a strategic intervention therapy, as demonstrated by preliminary results on this topic [82]. Stem cells, on the other hand, are able to reduce the number of recruited proinflammatory M1-type macrophages, while increasing the number of M2-type macrophages [83,84]. These two findings provide convincing evidence that stem-cell-mediated alteration of the immune compartment could represent a valuable target for further investigation. As described before, the effects mediated by direct cell injection are partially mediated by a paracrine effect. As demonstrated for other parameters, infusion of stem-cell-derived exosomes modulates macrophage populations. In particular, transcriptome data indicate that the enrichment of miR-181b in exosomes could mediate macrophage polarization by acting on its downstream target PKC*δ* [72].

Inflammatory modulation accompanies a positive effect on heart function parameters [85]. Using this information as starting point, a recent paper characterized the immune response after cell transplantation, underlining the importance of immune system activation, and in particular of macrophage polarization [86]. The authors compared the beneficial effects of two different stem cell populations (cardiac progenitor cells and bone marrow mononuclear cells, both extensively used in human clinical trials), non-viable cells (killed by freezing), and a chemical compound (zymosan) able to induce an immune response. Independently from what was injected, improved cardiac ventricular performance (estimated in term of fractional shortening) was observed. The treatment with immunosuppressants or the targeted depletion of macrophages inhibits the positive effects of the treatments, indicating a pivotal role of these cells during heart healing. An important point highlighted in this study is that the injection of both non-viable cells and zymosan is able to induce positive effects, indicating that neither paracrine effects nor cellular interaction are needed, but that activation of the innate immune system is essential to guiding heart regeneration.

## 6. Conclusions

**Figure 2:**
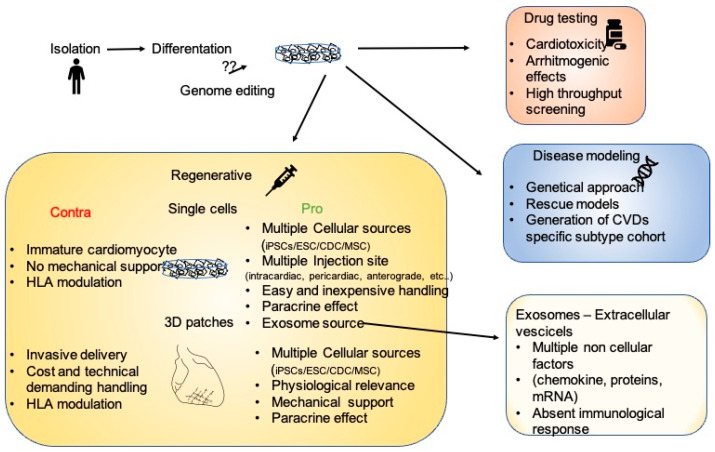
The many facets of iPSCs. After isolation and in vitro differentiation, iPSC cardiomyocytes could be employed as a platform for drug testing, for the modeling of multiple diseases allowing genetic characterization, or for regenerative therapies. In the regenerative scenario, the pros and cons of single cells or 3D structures are enumerated.

Stem cells are a powerful tool in fighting CVDs, and some light has now been shed on their mechanisms of action. Almost 20 years ago, the description of the unlimited potentiality of differentiation of embryonic stem cells gave an enormous impulse to the regenerative medicine field. From then on, when the in vitro differentiation of cardiomyocytes was the major challenge, methodological and intellectual progresses led to the development of highly sophisticated systems, such as patient-implantable cardiopatches. Even though iPSC applications have increased exponentially in recent years, from drug discovery platforms to clinically approved therapeutic treatments (Figure 2), some of the molecular mechanisms regulating iPSC biology are still debated. In particular, when analyzing the considerable amount of work describing the mechanisms regulating iPSC-mediated heart remodeling, the result is a highly complex, multifactorial picture, where cardiomyocytes exert paracrine and mechanical functions. In addition, interdisciplinary approaches led to the discovery that the innate immune system is another key player during heart remodeling. In a portrait where stem-cell-derived cardiomyocytes share the focus with cellular-derived factors and structural support, the immune system could be seen as the frame surrounding all other subjects. Due to this highly interconnected picture, and to the distinct characteristics of different CVDs, some questions need to be answered in order to determine patient/disease-specific optimal therapeutic approaches. Is an invasive transplantation of a 3D structure necessary? Or is a catheter-based delivery of extracellular vesicle/exosomes equally effective? Furthermore, how will the immunosuppression problem be solved? If the activation of innate immunity is fundamental, is the treatment of patients with cells that do not elicit any type of immune reaction recommended? Is the fine tuning of the immune system enough to drive an endogenous healing of the heart?

The answers to these highly complex questions call for a collaborative effort of specialized working groups with wide-ranging expertise in different research fields. In recent years, the foundation of multiple consortiums has already enabled the establishment of worldwide research alliance in order to join forces to analyze and translate stem cell therapeutic approaches into clinically approved treatments.

## Data Availability

Not applicable.
